# Human Curriculum Effects Emerge with In-Context Learning in Neural Networks

**Published:** 2024-02-13

**Authors:** Jacob Russin, Ellie Pavlick, Michael J. Frank

**Affiliations:** Department of Computer Science, Department of Cognitive, Linguistic, and Psychological Sciences, Brown University; Department of Computer Science, Carney Institute for Brain Science, Brown University; Department of Cognitive, Linguistic, and Psychological Sciences, Carney Institute for Brain Science, Brown University

**Keywords:** neural networks, blocking, large language models, metalearning, in-context learning

## Abstract

Human learning is sensitive to rule-like structure and the curriculum of examples used for training. In tasks governed by succinct rules, learning is more robust when related examples are blocked across trials, but in the absence of such rules, interleaving is more effective. To date, no neural model has simultaneously captured these seemingly contradictory effects. Here we show that this same tradeoff spontaneously emerges with “in-context learning” (ICL) both in neural networks trained with metalearning and in large language models (LLMs). ICL is the ability to learn new tasks “in context” — without weight changes — via an inner-loop algorithm implemented in activation dynamics. Experiments with pretrained LLMs and metalearning transformers show that ICL exhibits the blocking advantage demonstrated in humans on a task involving rule-like structure, and conversely, that concurrent in-weight learning reproduces the interleaving advantage observed in humans on tasks lacking such structure.

## Introduction

One of the most unique aspects of human behavior is its flexibility: humans can rapidly adapt to novel contexts or goals (Miller & Cohen, 2001), infer and apply arbitrary rules (Rougier, Noelle, Braver, Cohen, & O’Reilly, 2005), and plan far into the future (Botvinick & Weinstein, 2014; Frank & Badre, 2012). A key property thought to underlie this kind of cognitive flexibility is compositionality: novel concepts, expressions or plans can be understood as compositions of familiar ones, thereby allowing a potentially infinite number to be understood or deployed from only a limited set of learning experiences (Fodor & Pylyshyn, 1988).

Recent empirical results have offered a new context-sensitive perspective on human compositionality, revealing that it can be encouraged or discouraged by certain aspects of the learning task such as its *curriculum*, i.e., the order in which items are presented (Dekker, Otto, & Summerfield, 2022). In particular, human compositional generalization performance improves when related trials are blocked or correlated over time rather than interleaved or randomly shuffled over time. This kind of blocking advantage does not emerge in vanilla neural networks, but can emerge in those with specialized Hebbian gating mechanisms (Dekker et al., 2022; Flesch, Nagy, Saxe, & Summerfield, 2022) or those in which activation dynamics in prefrontal cortex are gated by reinforcement learning (Rougier et al., 2005).

These findings are consistent with studies on human category learning showing that humans exhibit a blocking advantage on tasks governed by rule-like structure (Ashby & Maddox, 2011). However, in the absence of such structure, the reverse effect, an interleaving advantage, is observed (Noh, Yan, Bjork, & Maddox, 2016). This pattern of results has been taken to support a dual-systems account, which posits a rule-based system that learns by hypothesis testing, and a procedural system that learns by integrating information over time (Ashby & Maddox, 2011; Noh et al., 2016). According to this account, the rule-based system operates by default in the presence of rule-like structure and benefits when trials are blocked, which eases the cognitive demands of the hypothesis-testing process. The procedural learning system can operate in the absence of rule-like structure, and shows an interleaving advantage because it benefits from the juxta-position of different exemplars over time.

Previous neural network models have separately reproduced the blocking and interleaving advantages. As noted above, in the presence of rule-like structure, neural networks with gated activation dynamics or learning can benefit from blocking (Dekker et al., 2022; Giallanza, Campbell, & Cohen, 2024; Rougier et al., 2005; Russin, Zolfaghar, Park, Boorman, & O’Reilly, 2022). Conversely, it has long been known that in the presence of interference, learning in neural networks benefits from interleaving (McClelland, McNaughton, & O’Reilly, 1995), which otherwise suffer from catastrophic forgetting (McCloskey & Cohen, 1989). However, no neural network model has simultaneously accounted for the full set of these curriculum effects, nor explained how such seemingly contradictory phenomena can coexist in a single system. Furthermore, previous models have been narrowly specialized, making it unclear whether their principles (e.g., Hebbian learning) would scale to the context of human-like cognitive flexibility in real-world environments.

Recently, deep neural networks such as large language models (LLMs) have achieved remarkable progress in their real-world capabilities (Brown et al., 2020), and are arguably the most cognitively flexible systems built to date (Bubeck et al., 2023). Much of this flexibility stems from their capacity to learn *in-context*, i.e., without any updates to their weights. To give one of these models a novel task, the user can simply supply explicit instructions or demonstrations, and the model will learn what to do from the context alone. In these settings, the model can be understood as implementing an *in-context learning* (ICL) algorithm in its forward dynamics, separate from the *in-weight learning* (IWL) algorithm used to train the network in the first place (Chan, Santoro, et al., 2022; Chan, Dasgupta, et al., 2022; Singh et al., 2023). This distinction between ICL and IWL has connections to human data and biologically motivated models of the interactions between working memory and reinforcement learning in humans, which emphasize the computational trade-offs that can occur in a single system equipped with both mechanisms (Frank & Claus, 2006; Collins & Frank, 2018; Rac-Lubashevsky, Cremer, Collins, Frank, & Schwabe, 2023).

In LLMs, the ICL algorithm emerges spontaneously in the course of training to continuously predict the next word on huge amounts of text (Brown et al., 2020; Xie, Raghunathan, Liang, & Ma, 2022), but neural networks can also be directly trained to implement an ICL algorithm via metalearning (Binz et al., 2023; Lake & Baroni, 2023; von Oswald, Niklasson, Randazzo, et al., 2023). In metalearning, a model is trained on a distribution of tasks so that it *learns how to learn* new tasks more efficiently or to generalize on new tasks in nontrivial ways (Wang et al., 2016, 2018; Wang, 2021). Lake and Baroni (2023) showed that metalearning can be used to train a network specifically to implement an ICL algorithm that captures human-level compositionality.

We hypothesized that neural networks capable of both ICL and IWL would reproduce the curriculum effects observed in humans (Dekker et al., 2022; Noh et al., 2016), with the blocking and interleaving advantages arising as consequences of ICL and IWL, respectively. We predicted that ICL would dominate in the presence of rule-like structure because the network would be capable of deciphering the simple rules governing the task via the inferential process taking place in its activation dynamics (Xie et al., 2022). A blocking advantage would arise in this case because this inferential process would be facilitated when related trials were blocked over time. We predicted that ICL would fail in the absence of simple rule-like structure, leading to more errors, which, when backpropagated to the network’s weights in the usual way, would result in more significant IWL. In this case, an interleaving advantage would arise because IWL would suffer from catastrophic forgetting when trials were blocked (McClelland et al., 1995; Russin et al., 2022).

In the following, Experiment 1 shows that in LLMs, ICL succeeds in the presence of rule-like structure and demonstrates a blocking advantage. Experiment 2 demonstrates in the metalearning setting that concurrent ICL and IWL in a single neural network reproduces the full spectrum of curriculum effects observed in humans.

### Task Design

**Figure 1:**
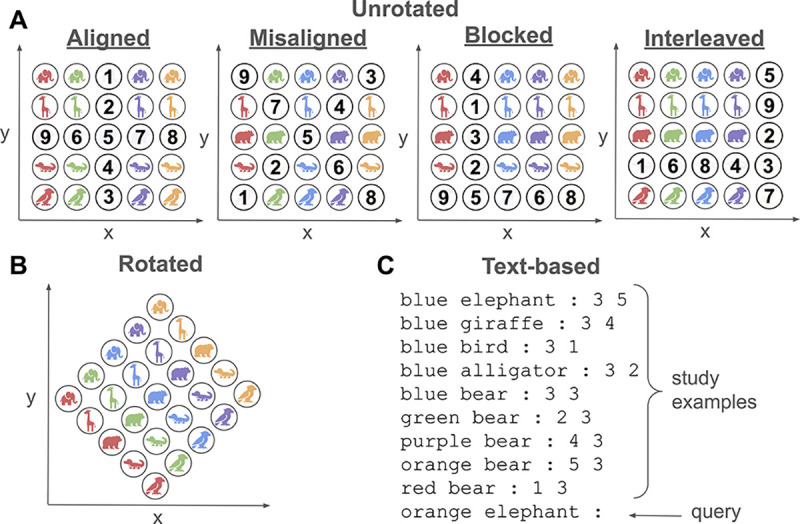
Compositional generalization task from Dekker et al. (2022) used in all experiments. **(A)** Curriculum conditions. The cues used as study examples and their presentation order are indicated by the overlaid numbers. **(B)** Rotated task inspired by Noh et al. (2016). **(C)** Text-based version.

All models were evaluated on a text-based version of the compositional generalization task used in Dekker et al. (2022). In the study, participants learned the reward locations corresponding to particular cues, which could be one of five different animals in one of five different colors (see Figure 1). Reward locations were systematic: one feature indicated the x-coordinate and the other indicated the y-coordinate. 9 of the 25 cues were used in “study examples” (i.e., training trials), where participants received feedback about the true reward location, and the other 16 were used in testing trials, where a given cue (“query”) was tested but no feedback was given.

The key manipulation affecting performance was the curriculum of examples studied before the generalization tests — which 9 cues were used as study examples, and the order in which they were presented (see Figure 1A). In the **Aligned** and **Blocked** conditions, but not in the **Misaligned** and **Interleaved** conditions, participants saw sequences (blocks) of cues that varied in one feature at a time (e.g., green giraffe, green alligator, green bear, …), thus facilitating any learning resembling a sequential inference or hypothesis-testing process, as has been theorized to occur in the human rule-based learning system (Noh et al., 2016), and in ICL (Xie et al., 2022). Indeed, Dekker et al. (2022) found that humans generalized to the test samples better in the Aligned condition than in the Misaligned condition, and better in the Blocked condition than in the Interleaved condition.

Noh et al. (2016) found the same blocking advantage in a similar rule-based task, which reversed to become an interleaving advantage when the feature space was rotated. To test whether the same interleaving advantage could be induced in the Dekker et al. (2022) task, we **Rotated** the color-animal grid by 45 degrees (see Figure 1B). This destroyed the rule-like structure of the task because a change along either feature no longer resulted in a simple change to the x or y coordinate, meaning it was no longer possible to infer a simple rule like ‘color=x, animal=y.’ We implemented both versions in a text-based form suitable for evaluating LLMs and metalearning neural networks, where the study examples were given *in context* with the query (see Figure 1C).

## Experiment 1: ICL in LLMs

We first explored whether LLMs, widely known to exhibit ICL (Brown et al., 2020), would reproduce the human blocking advantage on the text-based version of the task. In particular, we hypothesized that ICL would achieve better generalization performance in the aligned and blocked conditions than in the misaligned and interleaved conditions. Furthermore, we predicted that ICL would generalize well in the presence of rule-like structure (on the unrotated task), and poorly in the absence of such structure (on the rotated task).

### Models

We evaluated GPT-3.5 (Brown et al., 2020; Ouyang et al., 2022) and Llama 2 (Touvron et al., 2023) on the task. In GPT-3.5 (“gpt-3.5-turbo-instruct”), the temperature was set to 0.1, and five runs were performed. Llama 2, an open source model with approximately 70 billion parameters, was evaluated for one run using greedy decoding. A number of different prompts for each model were tried, but good performance was achieved with simple prompts containing only the study examples with no further instruction (see Figure 1C).

### Results

**Figure 2:**
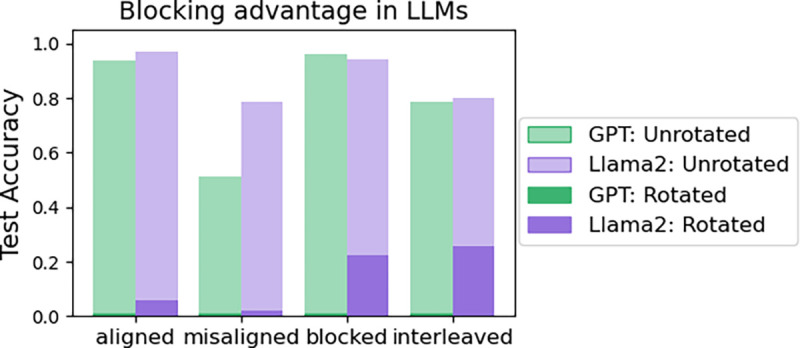
LLMs qualitatively reproduced the curriculum effects observed in humans, performing better when trials were aligned than misaligned, and better when trials were blocked than interleaved. When the task was rotated (darker colors), generalization performance dropped in all conditions.

Both LLMs qualitatively reproduced our hypothesized results. ICL in both models exhibited the **blocking advantage**: test performance was better in the aligned than misaligned condition, and in the blocked than interleaved condition (see Figure 2, light bars). ICL in both models also performed much worse when the task was rotated, generalizing poorly across all conditions (see Figure 2, dark bars).

These results were consistent with our hypotheses that in the presence of rule-like structure, ICL would perform well and could account for the blocking advantage. We also hypothesized that when ICL failed in the absence of such structure, more errors would be backpropagated, resulting in IWL and an interleaving advantage. Because of the cost associated with training LLMs, we chose to investigate this hypothesis in the metalearning setting.

## Experiment 2: ICL and IWL in Metalearning

To investigate the interplay between ICL and IWL within a single model, we adopted a metalearning approach. We trained neural networks from scratch on a distribution of compositional generalization problems based on the same task. The goal of this training was to reproduce in the same transformer architecture an ICL algorithm with similar properties to those observed in the LLMs, so that we could investigate its interaction with concurrent IWL. The metalearning approach afforded us full control over the model’s pretraining, allowing us to design a distribution of tasks that would impart inductive biases for an ICL algorithm with a preference for the blocked condition in the unrotated task.

Note that here our goal is not to explain the *origins* of these properties of ICL — we have already shown their spontaneous emergence in LLMs. Rather, the purpose of metalearning is to endow a network with ICL so that when it is presented with a new task it can be treated as analogous to a human participant who comes into an experiment equipped with a wealth of knowledge about how to learn in context (e.g., how to follow instructions or infer latent rules). This allows us to model the interaction between ICL and concurrent IWL that we hypothesize will reproduce the full set of curriculum effects observed in humans.

### Methods

#### Metalearning

Each task (“episode”) was randomly generated in the same way. First, a particular coordinate (1 through 5) was randomly assigned to each color and each animal. Then, the two cue features were randomly assigned to the two grid dimensions (i.e., color = x and animal = y, or vice versa). The 9 study examples to be given in context were then randomly chosen according to the blocked condition.

Each episode was constructed by concatenating a context string containing the 9 study examples, along with their true xy-coordinates, to a particular query for testing. The query could either be one of the 9 cues in the study examples (that were thus already present in the context), or one of the 16 other cues for testing compositional generalization. The metalearning training set consisted of 12,000 such episodes. 100 episodes were held out for validation and 10 episodes were held out for testing. These held-out episodes were not seen during training, thus ensuring that correct answers on test cues truly represented compositional generalization.

#### Finetuning

The usual form of learning in neural networks is IWL, but this metalearning procedure ensured that the model was also capable of ICL. The metalearned ICL algorithm is realized within its activation dynamics (i.e., in the flow of information from the inputs, containing the study examples and the query, to the output, which was a predicted reward location for the query). Thus, ICL can occur even when the network weights are frozen — even when no errors are backpropagated to update the weights. IWL, on the other hand, occurs precisely when the network weights were updated by backpropagating ICL errors.

During the **Few-shot** evaluation phase, the weights of the model were frozen and ICL was evaluated on held-out episodes, thus comprising a test of compositional generalization. During the **Finetuning** phase, the model was given a held-out episode, and could learn in context and/or in weights (by backpropagating any ICL errors). The structure of the samples was the same as during metalearning, but the model was only trained with queries that came from the cues in the study examples (thus emulating the experience of the participants, who only received feedback on the study examples).

To simulate the curriculum (e.g., blocked vs interleaved), we separated the 9 study examples into two groups based on which feature was varied: one group corresponded to a row in the grid, and one corresponded to a column (see Figure 1A). In the blocked condition, finetuning proceeded by training one block at a time — i.e., by training on one such group (‘TrainA’) for a fixed number of steps before switching to the other group (‘TrainB’). For example, a model might see samples only from one particular row of the grid for N steps, before seeing samples from one particular column for N steps.

Thus, in the blocked condition, samples were blocked in two distinct but congruent ways: 1) the study examples were blocked *over the context* (i.e., they were blocked in the context window), and 2) the samples were blocked *over the gradient steps* (i.e., the model was finetuned for a fixed number of gradient steps on samples containing queries from the TrainA group, then for a fixed number of steps on samples containing queries from the TrainB group, and so on). Likewise, in the interleaving condition, the samples were interleaved in two distinct but congruent ways: 1) the study examples were randomly shuffled over the context window, and 2) the samples were randomly shuffled over the gradient steps.

#### Model Details

We used the same transformer architecture (Vaswani et al., 2017) as Llama 2 (Touvron et al., 2023), but one that was much smaller and trained from scratch. Our model had 12 layers, 8 heads, a hidden size of 64 and a feed-forward size of 128, giving a total of 496,064 parameters.

#### Training Details

The metalearning (pretraining) and evaluation (finetuning) stages used different optimization settings. During pretraining, models were trained with a batch size of 256 and a learning rate of 0.001 using the Adam optimizer (Kingma & Ba, 2015) for up to 500 epochs with early stopping. During finetuning, models were trained with a batch size of 5 (batches were comprised of either one row or one column of the grid in the blocked condition) and a learning rate of 0.0001 with the Adam optimizer (Kingma & Ba, 2015) for 4 blocks and N=1000 steps per block.

### Results

**Figure 3:**
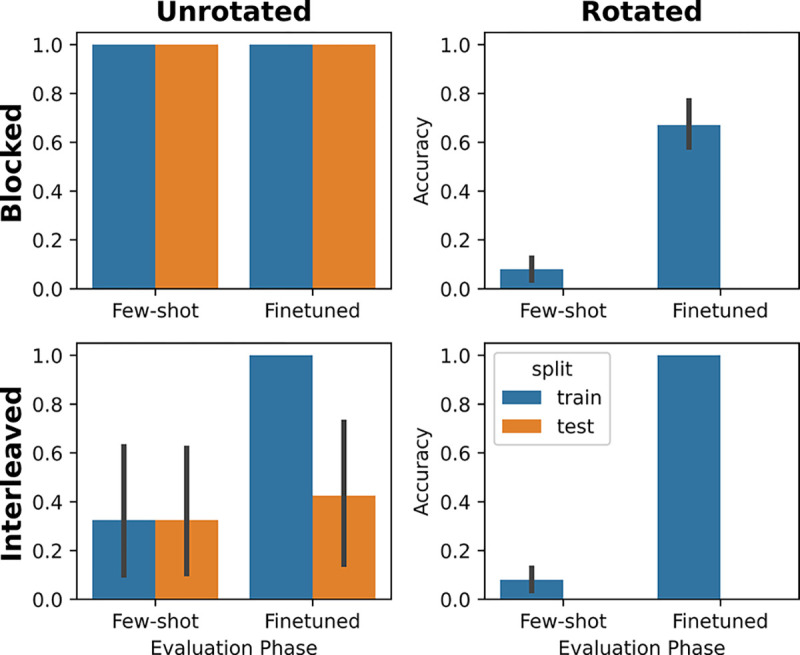
Metalearning results. In the unrotated task, ICL was successful, as shown by good few-shot test performance. ICL also showed a blocking advantage, performing better in the few-shot setting when trials were blocked than interleaved. In the rotated task, ICL was unsuccessful, as shown by poor few-shot performance. This led to greater IWL, which exhibited an interleaving advantage, as shown by better finetuning performance when trials were interleaved than when they were blocked. Error bars represent 95% confidence intervals.

When both ICL and IWL were active in a single network, the model recapitulated the full set of predicted curriculum effects (see Figure 3). In the unrotated task, when the model was tested in the few-shot setting, compositional generalization performance was better when trials were blocked compared to interleaved. This **blocking advantage** is perhaps unsurprising given the design of the metalearning dataset, where trials were always blocked over the context, but it is important to note that it still manifests in held out data requiring few-shot compositional generalization.

**Figure 4:**
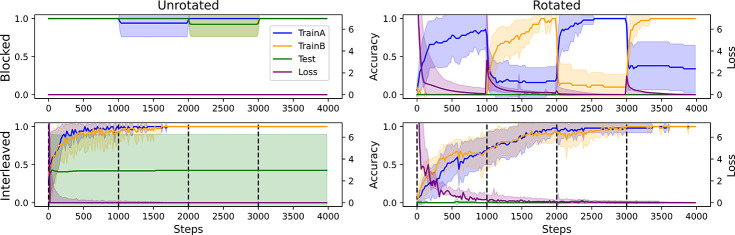
Detailed metalearning results. Each plot shows accuracy on samples trained in the first block (TrainA), the second block (TrainB), on test samples (Test), and the Loss. In the unrotated task (left), ICL succeeded and exhibited a blocking advantage, as shown by the perfect accuracy and near-zero loss when trials were blocked (top left), even before any finetuning had occurred. In the rotated task (right), ICL failed, causing greater IWL and leading to an interleaving advantage due to greater catastrophic forgetting when trials were blocked (top right). This can be seen in the drop in TrainA accuracy while finetuning on TrainB during the second block, and so on. No catastrophic forgetting occurs when trials are interleaved (bottom right).

The model’s ICL algorithm succeeded on the unrotated task when trials were blocked because these conditions were prevalent during metalearning, allowing the model to more easily recognize new instances of the rule-like structure. Thus, although IWL could always occur during finetuning in principle (the weights were not frozen), the network made few ICL errors in the unrotated task and little loss was incurred, thereby preventing IWL in practice. In contrast, in the rotated task, the model’s ICL algorithm failed to generalize, resulting in poor few-shot performance (see Figure 3, right side) and large losses (see Figure 4, right side). When these losses were backpropagated to drive IWL, we observed catastrophic forgetting, a phenomenon known to be pronounced when trials are blocked because learning in weights during one block will overwrite learning in overlapping weights that occurred during previous blocks (McClelland et al., 1995). This can be seen in the results from the blocked condition in the rotated task (see Figure 4, top right), where we observed large drops in accuracy on cues learned in the previous block during learning in each subsequent block.

Notably, these same principles can also explain the **interleaving advantage** observed in humans on tasks lacking rule-like structure (Noh et al., 2016). In particular, whenever ICL fails, the system transitions into an IWL regime where catastrophic forgetting becomes a more relevant dynamic. In this regime, the interleaving advantage arises because catastrophic forgetting in IWL can only be avoided when trials are interleaved. This phenomenon can be seen in Figure 4 (bottom right), where even though the task is rotated (so loss is high), the model still successfully learns in weights because trials are interleaved, avoiding catastrophic forgetting.

The coexistence of ICL and IWL in a single neural network can thus offer a novel explanation of the curriculum effects observed in human learning: 1) when ICL is possible in the presence of rule-like structure (unrotated task), a blocking advantage occurs because blocking makes ICL inference easier (as was observed in the LLMs). 2) When ICL is not possible in the absence of such rule-like structure (rotated task), IWL becomes necessary, leading to an interleaving advantage due to the increased relevance of catastrophic forgetting.

## Discussion

Many dual-systems theories posit a deliberative, controlled, or model-based system that is responsible for the most impressive aspects of human cognitive flexibility, and an unthinking, habitual, or model-free system with other advantages such as computational efficiency (Botvinick et al., 2019; Frank & Badre, 2012; Kahneman, 2011; Miller & Cohen, 2001; O’Reilly, Nair, Russin, & Herd, 2020). A common theme in these theories is to show how the presence of two distinct modules with different learning properties allows the system as a whole to leverage the advantages of each. For example, symbolic representations in classical systems naturally capture the principle of compositionality while neural networks are better equipped for handling high-dimensional and continuous domains, leading some to advocate for a neuro-symbolic hybrid approach (G. Marcus, 2020). Similarly, the rule-based system and procedural system posited in human category learning can explain how humans are capable of capitalizing on learning advantages when trials are either blocked or interleaved (Ashby & Maddox, 2011).

In this work, we show that the same kind of strategic duality can emerge in an integrated neural system capable of both in-context and in-weight learning. In particular, our results show how compositionality and its attendant curriculum-related phenomena can be seen as emergent properties of an ICL algorithm implemented in a network’s activation dynamics, separate from the usual learning occurring in its weights (Wang et al., 2018). This kind of ICL algorithm emerges spontaneously in LLMs trained to predict the next word in a large corpus of text (Brown et al., 2020), but can also be deliberately cultivated via metalearning (von Oswald, Niklasson, Schlegel, et al., 2023; Wang et al., 2018).

Although standard IWL in a neural network may not embody the inductive biases necessary for explaining human compositional behavior (Lake, Ullman, Tenenbaum, & Gershman, 2016; Lake & Baroni, 2018; G. F. Marcus, 1998), our results are consistent with recent findings (Lake & Baroni, 2023) suggesting that a neural network can nonetheless come to implement an ICL algorithm capable of human-like compositional generalization. This opens up the possibility that human compositionality can also be seen as a property of an emergent ICL algorithm, and suggests a novel perspective on a long-standing puzzle (McGrath, Russin, Pavlick, & Feiman, 2024; Russin, McGrath, Pavlick, & Frank, under review; Russin, McGrath, Williams, & Elber-Dorozko, in prep; Fodor & Pylyshyn, 1988).

In addition to demonstrating good compositional generalization performance, the emergent ICL algorithms in both LLMs and in our metalearning network also reproduced the curriculum effects observed in humans (Dekker et al., 2022; Noh et al., 2016). Without any special modification, the ICL algorithm that spontaneously emerges in LLMs succeeds in the presence of rule-like structure (on the unrotated task), and exhibits a blocking advantage, consistent with our hypothesis that ICL would benefit when related trials are blocked because this makes it easier to infer the relevant rules.

In humans, blocking is likely to make in-context inference easier because it reduces working memory load and interference (Noh et al., 2016; Russin et al., 2022). The transformer architecture (Vaswani et al., 2017) used by the LLMs does not have this kind of working memory capacity limitation. However, the nature of the LLMs training datasets (very large corpora of natural language text) may have engendered in them a related bias to prefer computations restricted to local subsequences. This would make inferences more likely to succeed when trials were blocked because related items would be closer together and inferences could take place more locally.

The ICL algorithms in the LLMs failed when the task was rotated with respect to the intuitive dimensions of ‘color’ and ‘animal’ (see Figure 2), destroying its rule-like structure (e.g., ‘color = x, animal = y’). This is compatible with our hypothesis that ICL would make more errors in the absence of rule-like structure, thus putting the system as a whole into a regime where IWL was more prominent. This latter finding is also reminiscent of models that explain why humans learn to retain stimulus-response associations far more robustly if they first acquired them under working memory load and thus experienced larger prediction errors needed for synaptic learning (Collins & Frank, 2018; Rac-Lubashevsky et al., 2023).

In the metalearning setting, we followed up on this idea and showed that when ICL makes more errors, more significant learning occurs in weights when these errors are backpropagated (see Figure 4). This dynamic bears a strong resemblance to data showing that humans more robustly remember learned associations when they could not use working memory to acquire them (Rac-Lubashevsky et al., 2023), and provides a natural explanation for the interleaving advantage because greater IWL leads to increased catastrophic forgetting when trials are blocked (McCloskey & Cohen, 1989). This is consistent with complementary learning systems theory (McClelland et al., 1995; O’Reilly, Bhattacharyya, Howard, & Ketz, 2011), which emphasizes that overlapping representations should engender interference when trials are blocked over time. In this work, we have extended these ideas to show how this property of IWL in neural networks can coexist with the properties of ICL that lead to the blocking advantage in learning contexts governed by simple rule-like structure.

Our work complements a number of previous neural network models that capture similar phenomena (Dekker et al., 2022; Giallanza et al., 2024; Rougier et al., 2005; Russin et al., 2022). Rougier et al. (2005) showed that the abstract representations necessary for flexible behaviors form in a model of gated prefrontal cortex (PFC) activations when related trials are blocked over time. Russin et al. (2022) showed that a PFC-like neural network augmented with a bias for active maintenance and gating exhibits a blocking advantage in a task designed to study cognitive map formation (Park, Miller, Nili, Ranganath, & Boorman, 2020). Similar ideas were also explored by Flesch et al. (2022), who showed that a blocking advantage emerges in a neural network augmented with a Hebbian mechanism on a similar task (Flesch, Balaguer, Dekker, Nili, & Summerfield, 2018). Dekker et al. (2022) then used a similar model to explain the blocking advantage observed on their compositional generalization task.

Our theoretical account of the blocking advantage in humans is largely consistent with these prior models, but has a number of benefits. First, the earlier models are important proofs of concept, but the specific principles they leverage have not been shown to scale to human-level cognitive flexibility. While transformers and LLMs are biologically and psychologically implausible in many ways (Bender, Gebru, McMillan-Major, & Shmitchell, 2021; McCoy, Yao, Friedman, Hardy, & Griffiths, 2023), LLMs have demonstrated human-like performance in many real-world cognitive domains (Bubeck et al., 2023), thus affording a unique opportunity for insight into any high-level principles or mechanisms that might promote such flexibility in general. Our work suggests that one such principle may relate to the dynamic interplay between in-context and in-weight learning. Second, our work emphasizes a novel perspective in which both compositionality and the blocking advantage can be seen as emergent properties of an ICL algorithm. This establishes a theoretical link between curriculum-related learning phenomena and the growing body of work exploring metalearning in cognition and artificial intelligence (Griffiths et al., 2019; Wang, 2021).

Finally, to our knowledge, our work is the first to demonstrate both a blocking advantage and an interleaving advantage in a single neural network model, and thus accounts for additional related phenomena observed in human category learning (Noh et al., 2016), but not addressed by previous models. This allows us to make specific predictions about the interactions between the mechanisms underlying these phenomena. For example, some results have shown that there is an initial bias toward the rule-based or hypothesis-testing system (Ashby & Maddox, 2011; Noh et al., 2016). Our proposal offers a novel explanation for this observation: initial learning is biased to take place in context because learning only occurs in weights when ICL makes errors. This basic dynamic between ICL and IWL is also consistent with biologically plausible models of working memory and reinforcement learning in prefrontal cortex and basal ganglia (Frank & Claus, 2006; Frank & Badre, 2012; Collins & Frank, 2018) — mechanisms that have also been proposed to mediate metalearning in humans (Wang et al., 2018).

In conclusion, our work builds on recent results (Lake & Baroni, 2023) showing that compositionality can emerge in neural networks as a property of an in-context learning algorithm. Furthermore, it shows that the duality between ICL and IWL offers a novel perspective on the curriculum effects observed in human learning. This novel perspective may offer further insights into the nature of human cognitive flexibility in general.
